# Epidemiology of multimorbidity in China and implications for the healthcare system: cross-sectional survey among 162,464 community household residents in southern China

**DOI:** 10.1186/s12916-014-0188-0

**Published:** 2014-10-23

**Authors:** Harry HX Wang, Jia Ji Wang, Samuel YS Wong, Martin CS Wong, Fang Jian Li, Pei Xi Wang, Zhi Heng Zhou, Chun Yan Zhu, Sian M Griffiths, Stewart W Mercer

**Affiliations:** JC School of Public Health and Primary Care, Faculty of Medicine, The Chinese University of Hong Kong, Shatin, New Territories, Hong Kong; General Practice and Primary Care, Institute of Health and Wellbeing, University of Glasgow, 1 Horselethill Road, Glasgow, G12 9LX UK; School of Public Health, Guangzhou Medical University, Guangzhou, 510182 P. R. China

**Keywords:** Multimorbidity, Epidemiology, Community medicine, Population household survey, Healthcare

## Abstract

**Background:**

China, like other countries, is facing a growing burden of chronic disease but the prevalence of multimorbidity and implications for the healthcare system have been little researched. We examined the epidemiology of multimorbidity in southern China in a large representative sample. The effects of multimorbidity and other factors on usual source of healthcare were also examined.

**Methods:**

We conducted a large cross-sectional survey among approximately 5% (N = 162,464) of the resident population in three prefectures in Guangdong province, southern China in 2011. A multistage, stratified random sampling was adopted. The study population had many similar characteristics to the national census population. Interviewer-administered questionnaires were used to collect self-report data on demographics, socio-economics, lifestyles, healthcare use, and health characteristics from paper-based medical reports.

**Results:**

More than one in ten of the total study population (11.1%, 95% confidence interval (CI) 10.6 to 11.6) had two or more chronic conditions from a selection of 40 morbidities. The prevalence of multimorbidity increased with age (adjusted odds ratio (aOR) = 1.36, 95% CI 1.35 to 1.38 per five years). Female gender (aOR = 1.70, 95% CI 1.64 to 1.76), low education (aOR = 1.26, 95% CI 1.23 to 1.29), lack of medical insurance (aOR = 1.79, 95% CI 1.71 to 1.89), and unhealthy lifestyle behaviours were independent predictors of multimorbidity. Multimorbidity was associated with the regular use of secondary outpatient care in preference to primary care.

**Conclusions:**

Multimorbidity is now common in China. The reported preferential use of secondary care over primary care by patients with multimorbidity has many major implications. There is an urgent need to further develop a strong and equitable primary care system.

**Electronic supplementary material:**

The online version of this article (doi:10.1186/s12916-014-0188-0) contains supplementary material, which is available to authorized users.

## Background

As the largest developing country in transition, chronic diseases have already become China’s most challenging health threat [1], accounting for 79% of all deaths, among which, cardiovascular diseases, cancer, chronic respiratory disease and diabetes contributed to approximately 33%, 20%, 17% and 1%, respectively [2]. Studies in western countries show that many people living with chronic disease have two or more (multimorbidity) [3]. A recent large, nationally representative study in Scotland demonstrated that across 40 chronic conditions, there were more people with multimorbidity than a single disease alone [4]. Multimorbidity is becoming the norm rather than the exception [5]. However, most guidelines are planned and implemented using a single disease approach in which diseases are treated in isolation [6]. Multimorbid patients are often treated by a range of different healthcare specialists (for each chronic disease), especially in China where specialist care is dominant [7]. This very often results in numerous different hospital visits, polypharmacy, repeated investigations and substantial treatment burden [8,9]. The over-reliance on secondary or tertiary-level care in countries with poorly developed or inequitable primary care systems also rapidly leads to care which is costly, duplicative and fragmented [10].

The demographic transformation in the aging structure is happening apace in China, where the proportion of older people 60-years old and more (12%) will exceed that of people 0- to 14-years old by 2019, and is expected to grow to approximately 34% of its total population by 2050 [11]. This will likely translate into substantial increases in the burden on health systems. Since 2009, China has stepped up its efforts to orient the healthcare system towards primary care [12-14]. To try to reduce the over-reliance on hospitals and to provide more equitable healthcare [15], community health centres (CHCs) are being set up in urban areas as primary care providers [16,17]. Unlike the UK [18], China’s primary care providers do not have a gate-keeper function, that is, referral from primary care doctors to hospital specialists (secondary care providers) is not mandatory. Patients can directly consult a doctor in primary care or secondary care. In addition, healthcare services are not free at the point of access. Although China has a social medical insurance system (which largely relies on monthly contributions from both employers and individuals), the benefit package is weak and service items covered are limited. Health care still largely relies on out-of-pocket payments, especially among those who are uninsured [19]. Thus, the affordability for patients (and their families), and the population’s perceptions towards healthcare providers may be important determinants of the use of primary and secondary care.

In developed countries, such as the UK, Canada, United States, Europe, Australia and Japan, the epidemiology of multimorbidity, its relationship to health service utilisation, and how it is affected by socio-economic status has been investigated [20,21]. However, such information is lacking in China. The current study describes the epidemiology of multimorbidity in a large, representative sample in southern China, and explores factors associated with multimorbidity and its association with the usual source of healthcare.

## Methods

China’s healthcare largely relies on paper-based medical records, albeit pilot initiatives are being made towards establishing a nation-wide electronic health record system. Thus, routine electronic healthcare data of the type used in previous studies in the West [4,22] are not currently available. A survey study design to collect self-report data combined with paper-based medical reports is, therefore, the most feasible way to examine the epidemiology of chronic conditions and their associations with the usual source of healthcare.

### Study design

We conducted a large cross-sectional community household survey (sponsored by the Department of Health, Guangdong province) among approximately 5% of the general resident population of all ages in three prefectures in Guangdong province, southern China in 2011. The prefecture setting in this study are medium-to-small scale cities or towns consisting of fifteen-to-thirty districts with total household population size of around one-to-two million. These prefectures have characteristics similar to the national average in terms of population demographics [23], urbanisation rate (40.11 *versus* 34.71) [24] and CHCs per unit population ratio (6.97 *versus* 5.74) [25]. CHCs that are government-owned and hospital-managed serve as the major primary care provider, which are regarded as a department within the hospital and typically function as an outreach clinic [17]. A multistage, stratified random sampling was adopted for selection of neighbourhood residential communities. Households within residential communities were then randomly selected from the household lists obtained from the Community Neighbourhood Authority (a grass-roots administrative agency). This Authority oversees the residential communities for household registration (also known as, ‘hukou’, an official identification of a person as a regular resident of an area). The number of households required was calculated using the standard formula adapted from an international guideline on designing household surveys [26]. The sampling framework is shown in detail in Additional file 1: Figure S1.

### Survey protocol and interviewer training

The questionnaire was derived from the National Health Services Survey (NHSS) 2008 [27] (which has been conducted every five years since 1993 and is overseen by the Center for Health Statistics and Information, Ministry of Health). We directly used the questions in the original NHSS to collect information on demographics, socio-economics, lifestyle behaviours and healthcare characteristics. The original close-ended question (consisting of fourteen chronic conditions) was modified into an open-ended question (‘Do you have any chronic conditions that have been diagnosed or treated by any healthcare providers within the past six months?’) and diseases were coded according to the International Classification of Diseases (ICD-10). In addition, we replaced ‘total household income’ with ‘household income per head’ which was used in our previous research [17] to take into account the household size. A panel consisting of two family medicine academics (FJL and ZHZ) and two public health professors (JJW and PXW) rated the relevancy and clarity of each questionnaire item and the content validity index was computed using a four-point Likert-type scale [28]. All items were rated as quite (three-point) or highly (four-point) relevant and clear by all panel experts to ensure the content validity. Four groups of medical students (10 students in each group) and healthcare staff at local CHCs were recruited as survey interviewers. Training workshops were held by JJW at Guangzhou Medical University. An interview manual was provided and practice sessions of mock interviews were arranged to improve inter-rater reliability. The questionnaire was pilot tested among all household members in 30 randomly selected households from 1 randomly selected residential community in each prefecture. Review sessions were held by JJW after every 10 household interviews to refine the questionnaire layout and wording. It was then tested in the subsequent pilot interviews to ensure all questions could be answered easily and without any ambiguity [see Additional file 2: Table S1].

### Data collection and fieldwork implementation

The interview groups conducted door-to-door surveys, and household replacement was made by targeting the next door on the left-hand side after three unsuccessful attempts. All residents with ‘hukou’ were invited for interview and migrants were excluded. For those who were absent from home at the time of visit (after two unsuccessful attempts) or those with cognitive difficulties, information was gathered from the householder or the guardian, whoever was most familiar with him/her. All respondents who self-reported the presence of chronic conditions were invited to examine their paper-based medical reports obtained from previous healthcare visits and annual check-ups to reduce recall bias. Conditions that were not reported by the respondents were reviewed by onsite healthcare staff to supplement the information provided. Each completed questionnaire was checked for correctness by one on-site researcher, and suspect cases were re-surveyed. Data entry was conducted by two trained university students independently, and double entry verification was performed using EpiData software version 3.1 (Denmark) [29] to improve data accuracy.

### Morbidity coding

All chronic conditions were reviewed by the research panel. The selection of included morbidities was based on the methodology adopted in a previous UK study [4] and another systematic review [30] in which morbidities recommended as a core for international multimorbidity studies were listed. To take into consideration China’s healthcare context, major morbidities captured in the National Health Services Survey in China [27] were also included. A total of 40 chronic conditions [see Additional file 3: Table S2] were selected after panel review and rare chronic conditions were excluded. All chronic conditions were weighted equally according to other international studies [4,31].

### Statistical analysis

The Chi-square goodness-of-fit test was applied to compare characteristics of the study population and national census population to provide information on non-coverage error. The average numbers of morbidities across groups were compared using Student’s t-test or one-way analysis of variance (ANOVA), when appropriate. Binary logistic regression analysis was conducted to examine factors associated with multimorbidity and healthcare utilisation outcomes after controlling for demographic and socio-economic confounders. A backward stepwise algorithm was used to explore independent variables. The absence of multicollinearity and plausible interactions among variables were tested to ensure the robustness of the regression model. Differences were regarded as statistically significant if *P* values were less than 0.05. All statistics were calculated by using base weights (proportional to population size) with post-stratification sample weights adjustment (based on the demographic estimates from the national census [23]) to increase the representativeness of the study population. To account for the multistage sample design, statistical analyses were performed using the Complex Samples module in IBM SPSS Statistics 20.0 (Chicago, IL, USA).

### Ethics statement

The study was approved by the Survey and Behaviour Research Ethics Committee of the Chinese University of Hong Kong and the Research Ethics Committee of Guangzhou Medical University.

## Results

**Figure 1:**
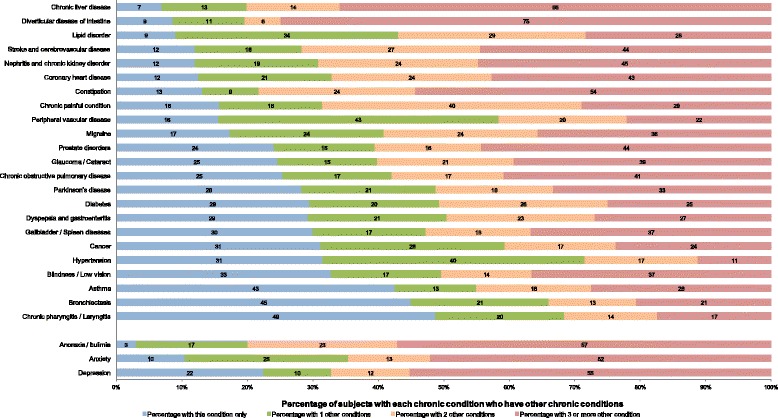
**Number of chronic conditions experienced by patients with common, important diseases.**

A total of 162,464 residents of all ages (4.55% of the general residents) from 53,760 households were included in the study [see Additional file 1: Figure S1]. The household replacement rate was 9.91% and 14.46% of total surveys were answered by householders/guardians on behalf of household members. The socio-demographic, lifestyle, and morbidity characteristics of all study participants are shown in Table 1. Compared to the national census population [23], the study population was slightly more educated (63.9% *versus* 61.75% for secondary school and above) [see Additional file 4: Table S3]. Overall, more than one in ten (11.1%, 95% confidence interval (CI) 10.6 to 11.6) of the study population had multimorbidity (Table 1). For people with any of the 40 chronic conditions examined, most had one or more other conditions rather than the single-condition alone (Figure 1).

**Table 1:** **Socio-demographic, lifestyle and morbidity characteristics of all study participants**

**Variables**	**Total (%)**	**Mean number of morbidities (SD)**	***P*** **value** ^**a**^	**Percentage with ≥1 morbidity (95% ** **CI)** ^**b**^	***P*** **value** ^**c**^	**Percentage with ≥2 morbidities (95%** **CI)** ^**b**^	***P*** **value** ^**c**^
**All participants**	162,464 (100.0%)	0.45 (1.00)		23.8% (23.0 to 24.6)		11.1% (10.6 to 11.6)	
**Gender**							
Female	78,972 (48.6%)	0.48 (1.07)	<0.001	22.8% (22.1 to 23.6)	<0.001	13.0% (12.4 to 13.6)	<0.001
Male	83,492 (51.4%)	0.42 (0.94)		24.7% (23.8 to 25.6)		9.2% (8.8 to 9.7)	
**Age, years**							
0 to 24	49,413 (30.4%)	0.06 (0.32)	<0.001	5.0% (4.7 to 5.2)	<0.001	0.9% (0.8 to 1.0)	<0.001
25 to 44	55,402 (34.1%)	0.20 (0.58)		14.3% (13.5 to 15.2)		3.7% (3.5 to 3.9)	
45 to 64	44,020 (27.1%)	0.80 (1.25)		40.4% (39.5 to 41.3)		20.5% (19.7 to 21.3)	
≥65	13,629 (8.4%)	1.74 (1.54)		76.9% (76.0 to 77.9)		47.5% (45.7 to 49.4)	
**Monthly household income per head**							
Less than ¥1,000	59,202 (36.9%)	0.43 (0.97)	<0.001	22.8% (22.1 to 23.5)	<0.001	10.4% (9.9 to 10.9)	<0.001
¥1,000 to 1,999	38,387 (23.9%)	0.45 (1.00)		24.5% (23.8 to 25.3)		10.8% (10.4 to 11.2)	
¥2,000 to 2,999	46,613 (29.0%)	0.47 (1.04)		24.2% (23.3 to 25.2)		12.0% (11.4 to 12.6)	
¥3,000 and above	16,392 (10.2%)	0.50 (1.06)		25.7% (24.6 to 26.7)		12.9% (12.2 to 13.6)	
**Marital status**							
Single	35,111 (21.9%)	0.16 (0.57)	<0.001	10.1% (9.6 to 10.6)	<0.001	3.7% (3.5 to 4.0)	<0.001
Married	119,675 (74.5%)	0.52 (1.07)		27.4% (26.6 to 28.3)		13.0% (12.4 to 13.6)	
Divorced	1,239 (0.8%)	0.37 (0.86)		21.6% (20.7 to 22.5)		8.8% (8.1 to 9.5)	
Widowed	4,570 (2.8%)	0.89 (1.42)		39.6% (38.4 to 40.9)		23.2% (21.9 to 24.6)	
**Education level**							
No education	18,876 (11.8%)	0.93 (1.33)	<0.001	45.8% (44.6 to 47.1)	<0.001	24.1% (23.0 to 25.4)	<0.001
Primary school	39,023 (24.3%)	0.58 (1.13)		29.4% (28.6 to 30.4)		14.4% (13.8 to 15.1)	
Secondary school	81,779 (50.9%)	0.33 (0.86)		18.5% (17.7 to 19.2)		7.8% (7.4 to 8.2)	
College and above	20,918 (13.0%)	0.27 (0.78)		15.3% (14.6 to 16.0)		6.9% (6.6 to 7.2)	
**Employment status**							
Unemployed	27,994 (17.4%)	0.70 (1.22)	<0.001	35.0% (33.7 to 36.4)	<0.001	17.8% (17.0 to 18.7)	<0.001
Employee	101,020 (62.9%)	0.32 (0.82)		18.6% (18.0 to 19.2)		7.3% (7.0 to 7.5)	
Retired	16,346 (10.2%)	1.18 (1.48)		53.6% (52.1 to 55.0)		31.8% (30.1 to 33.6)	
Student	15,235 (9.5%)	0.12 (0.51)		7.4% (7.0 to 7.8)		3.0% (2.9 to 3.2)	
**Medical insurance**							
Uninsured	25,705 (16.0%)	0.55 (1.07)	<0.001	28.5% (27.1 to 29.9)	<0.001	14.9% (14.3 to 15.6)	<0.001
Insured	134,890 (84.0%)	0.43 (0.99)		23.1% (22.3 to 23.8)		10.5% (10.0 to 11.0)	
**Usual source of healthcare**							
Primary level	100,903 (62.8%)	0.41 (0.96)	<0.001	22.0% (21.4 to 22.7)	<0.001	10.0% (9.6 to 10.4)	<0.001
Secondary/Tertiary level	53,601 (33.4%)	0.52 (1.07)		27.0% (26.0 to 28.1)		13.3% (12.5 to 14.1)	
Mixed/Not sure	6,091 (3.8%)	0.52 (1.06)		28.2% (25.9 to 30.5)		12.4% (11.8 to 13.1)	
**Hospitalisation**							
No	153,629 (94.6%)	0.39 (0.91)	<0.001	21.6% (20.8 to 22.4)	<0.001	9.7% (9.2 to 10.2)	<0.001
Yes	8,835 (5.4%)	1.46 (1.71)		62.3% (61.8 to 62.8)		35.0% (34.2 to 35.7)	
**Smoking**							
Non-smoker	134,036 (83.5%)	0.35 (0.89)	<0.001	19.0% (18.3 to 19.7)	<0.001	8.5% (8.0 to 8.9)	<0.001
Smoker	24,616 (15.3%)	0.99 (1.34)		49.7% (48.7 to 50.7)		25.6% (24.7 to 26.5)	
Ever-smoker	1,943 (1.2%)	0.78 (1.29)		39.1% (38.5 to 39.6)		17.9% (17.4 to 18.4)	
**Alcohol consumption**							
Seldom-drinker	141,482 (88.1%)	0.37 (0.92)	<0.001	20.2% (19.5 to 20.9)	<0.001	9.0% (8.6 to 9.4)	<0.001
Regular drinker	18,988 (11.8%)	1.04 (1.35)		51.6% (50.7 to 52.6)		27.5% (26.5 to 28.5)	
Ever-drinker	125 (0.1%)	1.40 (1.84)		55.5% (52.4 to 58.6)		32.4% (29.6 to 35.3)	
**Dietary preference**							
Normal	126,799 (79.0%)	0.45 (1.00)	<0.001	24.2% (23.4 to 25.1)	<0.001	11.2% (10.7 to 11.8)	<0.001
Salty diet	14,170 (8.8%)	0.73 (1.28)		34.9% (33.7 to 36.1)		19.1% (18.2 to 20.1)	
Bland diet	19,626 (12.2%)	0.25 (0.76)		14.1% (14.1 to 14.2)		5.4% (5.4 to 5.5)	
**Physical activity**							
No	65,483 (40.8%)	0.50 (1.06)	<0.001	25.3% (24.4 to 26.2)	<0.001	13.1% (12.5 to 13.8)	<0.001
Yes	95,112 (59.2%)	0.42 (0.97)		23.0% (22.2 to 23.7)		9.9% (9.4 to 10.3)	
**Number of chronic conditions**							
0	123,778 (76.2%)						
1	20,699 (12.7%)						
2	8,184 (5.0%)						
3	5,839 (3.6%)						
4	2,432 (1.5%)						
5	856 (0.5%)						
6	414 (0.3%)						
≥7	262 (0.2%)						

^a^Differences between means within each variable. *t* test for independent samples for gender, medical insurance, hospitalisation, physical activity; one-way analysis of variance (ANOVA) for age, monthly household income per head, marital status, education level, employment status, usual source of healthcare, smoking, alcohol consumption, and dietary preference; ^b^row percentages derived from the total number in the corresponding row; ^c^differences between categories within each variable. Chi-square test for 2 × n tables. CI, confidence interval; SD, standard deviation.

**Figure 2:**
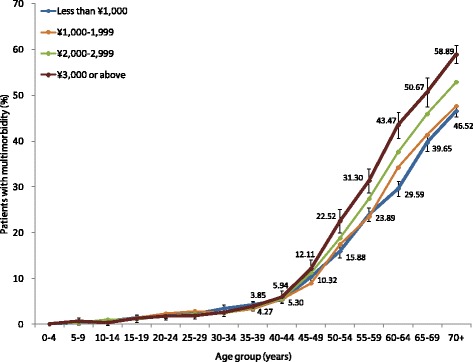
**Prevalence of multimorbidity by age and household income per head.** Note: Error bars indicate 95% confidence interval.

The number of morbidities and the proportion of people with multimorbidity increased substantially with age [see Additional file 5: Figure S2]. By age 55 years, half of the population had at least one morbidity, and by age 70 years, the majority was multimorbid. Logistic regression analysis with multimorbidity as the outcome showed that in addition to age, the factors most strongly and independently associated with multimorbidity were female gender, low education, unemployment, lack of medical insurance and lifestyle factors (smoking, alcohol drinking, salty diet and physical inactivity) (Table 2). People with higher per capita household income tended to report slightly more multimorbidity (Figure 2).

**Table 2:** **Associations between multimorbidity and age, gender, socio-economic and lifestyle behaviour factors**

**Covariates**	**Unadjusted OR**	**95%** **CI**	***P*** **value** ^**a**^	**Adjusted OR** ^**b**^	**95%** **CI**	***P*** **value** ^**a**^
**Age, per five years**	1.42	1.40 to 1.43	<0.001	1.36	1.35 to 1.38	<0.001
**Gender, female**	1.48	1.46 to 1.49	<0.001	1.70	1.64 to 1.76	<0.001
**Monthly household income per head**						
Less than ¥1,000	1.00 (Ref)		<0.001	1.00 (Ref)		<0.001
¥1,000 to 1,999	1.05	1.03 to 1.06		0.99	0.98 to 1.01	
¥2,000 to 2,999	1.18	1.15 to 1.20		1.09	1.08 to 1.11	
¥3,000 and above	1.28	1.24 to 1.32		1.15	1.12 to 1.18	
**Marital status**						
Single	1.00 (Ref)		<0.001	1.00 (Ref)		<0.001
Married	3.84	3.63 to 4.07		0.47	0.43 to 0.53	
Divorce	2.49	2.31 to 2.68		0.88	0.81 to 0.96	
Widowed	7.79	7.15 to 8.49		0.94	0.86 to 1.03	
**Education level**						
No education	1.00 (Ref)		<0.001	1.00 (Ref)		<0.001
Primary school	0.53	0.50 to 0.56		0.79	0.76 to 0.83	
Secondary school	0.27	0.25 to 0.28		0.69	0.66 to 0.72	
College and above	0.23	0.22 to 0.24		0.66	0.62 to 0.69	
**Employment status**						
Unemployed	1.00 (Ref)		<0.001	1.00 (Ref)		<0.001
Employee	0.36	0.34 to 0.38		0.62	0.59 to 0.65	
Retired	2.15	2.04 to 2.27		1.18	1.13 to 1.23	
Student	0.14	0.14 to 0.15		0.63	0.56 to 0.72	
**Medical insurance**						
Insured	1.00 (Ref)		<0.001	1.00 (Ref)		<0.001
Uninsured	1.49	1.44 to 1.55		1.79	1.71 to 1.89	
**Usual source of healthcare**						
Primary level	1.00 (Ref)		<0.001	1.00 (Ref)		<0.001
Secondary/Tertiary level	1.38	1.32 to 1.45		1.21	1.16 to 1.27	
Mixed/Not sure	1.28	1.24 to 1.31		1.20	1.15 to 1.25	
**Smoking**						
Non-smoker	1.00 (Ref)		<0.001	1.00 (Ref)		<0.001
Smoker	3.72	3.64 to 3.79		3.07	3.00 to 3.14	
Ever-smoker	2.36	2.23 to 2.49		1.92	1.80 to 2.04	
**Alcohol consumption**						
Seldom-drinker	1.00 (Ref)		<0.001	1.00 (Ref)		<0.001
Regular drinker	3.83	3.75 to 3.91		3.25	3.18 to 3.33	
Ever-drinker	4.84	4.30 to 5.45		3.49	3.07 to 3.97	
**Dietary preference**						
Normal	1.00 (Ref)		<0.001	1.00 (Ref)		<0.001
Salty diet	1.88	1.81 to 1.94		1.97	1.90 to 2.03	
Bland diet	0.45	0.43 to 0.48		0.49	0.47 to 0.51	
**Physical activity**						
Yes	1.00 (Ref)		<0.001	1.00 (Ref)		<0.001
No	1.38	1.33 to 1.43		1.31	1.27 to 1.35	

^a^ *P* values are based on joint tests, which test the overall differences between the individual categories of the corresponding variable; ^b^adjusted for all other covariates (independent variables) listed in the table. CI, confidence interval; OR, odds ratio; Ref, reference. Dependent variable: presence of multimorbidity (1 = Yes; 0 = No).

**Figure 3:**
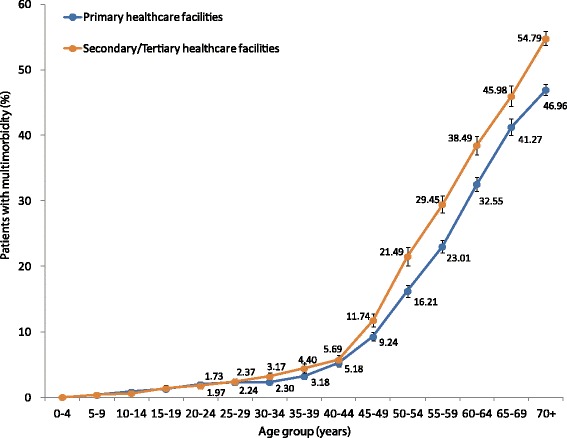
**Prevalence of multimorbidity by age and usual source of healthcare.** Note: Error bars indicate 95% confidence interval.

Multimorbidity was associated with the choice of usual source of healthcare services. Of the 154,504 subjects who had a usual source of healthcare, one third (34.7%, (53,601/154,504)) reported using outpatient services at secondary care regularly for tackling chronic diseases compared with 65.3% (100,903/154,504) using primary care in the past 12 months. At most ages, people for whom outpatient secondary care was their usual source of health care had a higher crude prevalence of multimorbidity (Figure 3). In the binary logistic regression model among all study participants, having chronic conditions, higher household income, higher education level and lack of medical insurance were independent factors significantly associated with using secondary outpatient care over primary care as usual source of healthcare. A similar pattern of usual source of healthcare was also shown among subjects with multimorbidity (Table 3).

**Table 3:** **Association between use of primary care facilities as usual source of healthcare and age, gender, socio-economic characteristics and morbidity factors**

	**All participants (Number = 162,464)**						**Participants with multimorbidity (Number = 17,988)**					
**Covariates**	**Unadjusted OR**	**95%** **CI**	***P*** **value** ^**a**^	**Adjusted OR** ^**b**^	**95%** **CI**	***P*** **value** ^**a**^	**Unadjusted OR**	**95%** **CI**	***P*** **value** ^**a**^	**Adjusted OR** ^**b**^	**95%** **CI**	***P*** **value** ^**a**^
**Age, per five years**	0.98	0.98 to 0.98	<0.001	0.99	0.99 to 0.99	0.008	0.98	0.98 to 0.98	<0.001	0.99	0.99 to 0.99	0.033
**Gender, male**	1.03	1.03 to 1.04	<0.001	1.05	1.04 to 1.05	<0.001	1.00	0.99 to 1.01	0.787	1.04	1.02 to 1.05	<0.001
**Monthly household income per head**												
Less than ¥1,000	1.00 (Ref)		<0.001	1.00 (Ref)		<0.001	1.00 (Ref)		<0.001	1.00 (Ref)		<0.001
¥1,000 to 1,999	0.74	0.74 to 0.75		0.74	0.74 to 0.75		0.92	0.91 to 0.94		0.90	0.88 to 0.91	
¥2,000 to 2,999	0.62	0.62 to 0.63		0.67	0.67 to 0.68		0.63	0.60 to 0.66		0.68	0.65 to 0.71	
¥3,000 and above	0.43	0.42 to 0.43		0.54	0.53 to 0.54		0.42	0.40 to 0.43		0.53	0.51 to 0.55	
**Education level**												
No education	1.00 (Ref)		<0.001	1.00 (Ref)		<0.001	1.00 (Ref)		<0.001	1.00 (Ref)		<0.001
Primary school	0.96	0.95 to 0.97		0.90	0.89 to 0.92		0.83	0.79 to 0.87		0.85	0.82 to 0.89	
Secondary school	0.81	0.80 to 0.82		0.69	0.68 to 0.70		0.63	0.60 to 0.66		0.66	0.62 to 0.69	
College and above	0.41	0.40 to 0.42		0.35	0.35 to 0.36		0.35	0.34 to 0.37		0.40	0.39 to 0.41	
**Employment status**												
Unemployed	1.00 (Ref)		<0.001	1.00 (Ref)		<0.001	1.00 (Ref)		<0.001	1.00 (Ref)		<0.001
Employee	0.94	0.93 to 0.95		1.10	1.09 to 1.10		0.92	0.89 to 0.95		1.04	1.01 to 1.08	
Retired	0.48	0.47 to 0.49		0.51	0.50 to 0.52		0.48	0.45 to 0.51		0.53	0.50 to 0.56	
Student	1.08	1.06 to 1.09		1.30	1.28 to 1.32		0.87	0.85 to 0.90		1.30	1.27 to 1.34	
**Medical insurance, insured**	1.43	1.41 to 1.45	<0.001	1.38	1.37 to 1.40	<0.001	1.26	1.23 to 1.29	<0.001	1.27	1.24 to 1.30	<0.001
**Number of chronic conditions**												
0	1.00 (Ref)		<0.001	1.00 (Ref)		<0.001	…			…		
1	0.82	0.80 to 0.85		0.84	0.82 to 0.86		…			…		
2	0.69	0.67 to 0.72		0.79	0.77 to 0.82		1.00 (Ref)		0.219	1.00 (Ref)		0.098
3	0.71	0.67 to 0.76		0.79	0.75 to 0.84		1.03	0.97 to 1.08		1.00	0.96 to 1.05	
≥4	0.71	0.65 to 0.78		0.82	0.76 to 0.89		1.02	0.94 to 1.11		1.05	0.98 to 1.12	

^a^ *P* values are based on joint tests, which test the overall differences between the individual categories of the corresponding variable; ^b^adjusted for other independent variables including age, gender, household income per head, education, employment, medical insurance, and number of chronic conditions. CI, confidence interval; OR, odds ratio; Ref, reference. Dependent variable: use of primary care facilities as usual source of healthcare (1 = Yes; 0 = No).

## Discussion

### Statement of principal findings

The present study investigated the prevalence of multimorbidity in a large representative sample in southern China. We have found that multimorbidity is common, increases with age, and that the majority of people with any chronic disease have one or more additional conditions. In addition to increasing age, female gender, low education, unemployment, lack of medical insurance and unhealthy lifestyles were factors independently associated with multimorbidity. Secondary care was more likely, and primary care less likely, to be used as usual source of healthcare among people with multiple chronic conditions, compared to those with no multimorbidity.

### Relationship with other studies

A large body of cross-sectional studies conducted in western developed countries has examined the epidemiology of multimorbidity [21]. Multimorbidity has been defined and assessed by various approaches, with diseases count per individual (as used in the current study) being the most common [32]. Estimates of the prevalence of multimorbidity vary widely in different studies in countries, depending on a number of factors including the age groups included, the sampling frame and the number of conditions included. The prevalence reported in the current study is commensurate with the ranges found in other countries [21], though somewhat lower than in most other large studies [4,21]. Whether this is a true difference between China and western countries or a reflection of different methods of estimating multimorbidity will require future studies specifically designed to examine this.

The higher prevalence of multimorbidity in women in the current study concurs with most of the previous literature [21]. The reason for this is not yet clear, and a range of factors may be at play [33,34]. The large effect of increasing age on the prevalence of multimorbidity was unsurprising, as numerous studies across the world have established this [3,21,35]. Age-related multimorbidity has major financial and social implications globally, as populations are rapidly ageing in most countries, including developing countries and those in transition. Multimorbidity impairs quality of life and functional ability, leading to frailty and dependency and massively escalating healthcare costs. Indeed, the burden of chronic disease is the biggest financial challenge to countries and healthcare systems world-wide. China, however, has an especially rapidly ageing population, as a result of not only improved longevity but also due to the one-child policy introduced in 1979 [36]. Estimates suggest a drastic decline in the older person-support ratio from 9 working-age adults (15- to 64-years old) per older person (65 years old and older) to only 2.5 by 2050 [37], that is, there will be far fewer working-age adults to support a rapidly ageing population. This may endanger the affordability of care in China as health care still largely relies on out-of-pocket payments [19]. The recent relaxation in the one-child policy [38] may allow the health care needs of the older population to be shared among more siblings and thus alleviate the burden on individuals in the long term. However, the high prevalence of multimorbidity in older people found in the present study is likely to challenge this, especially given the current preference for secondary care which is likely to be costly and duplicative [4].

The relationship between lifestyle factors and individual chronic conditions (such as obesity and type 2 diabetes) is, of course, well established, but the relationship with the co-occurrence of multiple long-term conditions has not been fully explored [39]. A recent Canadian study found a bivariate association between smoking and the prevalence of multimorbidity, but a lack of association with physical activity or alcohol consumption [40]. In contrast, our study showed an association between unhealthy lifestyle factors, including smoking, alcohol drinking, salty diet and physical inactivity and multimorbidity. The between-study variance might be due to the measurement of multimorbidity which only included fourteen frequent conditions in the Canadian study [40]. Nevertheless, both studies imply that promoting healthy lifestyles as a prevention and intervention strategy is likely to be important in the management of multimorbidity.

In contrast with the findings in western countries [4,21,22,41], our study shows that self-reported multimorbidity is associated with slightly higher household income per head in China. This association is attenuated but not eliminated by accounting for other socio-demographic covariates. This apparent paradox may be explained by the rapid escalation of medical care costs in China over the past decades [42], during which people with lower income have lower rates of diagnosed conditions due to unaffordablility and inadequate use of healthcare [43]. It may also reflect the phenomenon of ‘disease of affluence’ due to unhealthy lifestyle changes in some of the more affluent brackets within countries in transition, and has been reported in single-disease studies in China and elsewhere [44-47]. Further work is required to clarify this.

A generalist primary care-based approach has been suggested most appropriate for most multimorbid patients as it provides continuity and coordination of care [4]. Better continuity of care for those with chronic diseases may ultimately lead to lower episode-based costs, fewer hospitalisations and emergency department visits and fewer complications [48]. Recent work from Brazil has found that implementation of a nation-wide primary care approach has resulted in substantial reductions in morbidity and mortality from chronic diseases [49]. Fragmentation of health care in China is common, and continuity of primary care is often lacking [7]. The growth of hospital specialist care during the past twenty years has widened the divide between primary care and secondary care [16]. Although China is encouraging the utilisation of primary care by giving insured patients preferential rates, those uninsured or with a higher income, as shown in our study, appear to preferentially seek services directly at secondary care. This might reflect the fact that healthcare delivery in China is still dominated by secondary care [50], and specialists are often considered more trustworthy and skilful than general practitioners [51]. However, in other countries it has been found that multimorbid patients who rely on specialist services in secondary care have more difficulties with fragmentation of care [52]. Unlike those countries with strong primary care systems, such as the UK, primary care is still weak in China [16,51], and needs a properly trained and adequately resourced primary care system – an aspiration of China’s current healthcare reform [12-15]. Given that primary care is currently under-utilised by patients with multimorbidity, strengthening of access to, and trust in, primary care providers is required in order to enable primary care providers to lead the management of chronic conditions [1,14]. Progress toward the expansion of medical insurance coverage [53] should be accelerated as it has been shown to contribute to better primary care experience [17]. Initiatives to establish a general practitioner-based multidisciplinary team approach equipped with skilled healthcare professionals led by local government would then help attract and retain patients at the primary care level [17].

### Strengths and weaknesses of the study

This is the first large scale study to examine the epidemiology of multimorbidity across a wide range of chronic conditions and to explore its impact on healthcare preference in a large representative sample of the Chinese population. We gathered data on a large population who had many similar characteristics to the national census population, and we followed the most commonly used international definition of multimorbidity [3,4,31,54] in our study to increase the compatibility with the international literature. One of the major limitations of the study is the reliance on self-report of chronic diseases diagnosed by various healthcare providers, and we were unable to construct a criterion standard for rigid validation due to the absence of an electronic medical record system in China. Thus, the possibility of under-diagnosis or misclassification of diseases cannot be ruled out. However, any list of conditions that could be feasibly collected in a survey will inevitably be incomplete. Our morbidity count included morbidities widely used in previous Chinese research and conditions recommended as core for multimorbidity studies by a systematic review [30]. The weaknesses also include the cross-sectional nature of the study and, therefore, a cause-and-effect relationship could not be established. Last but not least, although we used data from a very large population whose characteristics were similar to the Chinese population as a whole, the study was conducted in just one region in southern China, and the south, in general, has a slightly higher urbanisation rate (lower rurality) and CHCs per unit population ratio (higher primary care service capacity) than the north. Thus, it is likely that the population in the north will generally have lower income (as there is more poverty in the rural areas than in the urban [23]) and less access to healthcare. Accordingly, the effect of socio-economic status on multimorbidity and the patterns of the regular use of secondary outpatient care over primary care for multimorbidity that we observed in this study might be starker in the north.

### Unanswered questions and future research

The variable of health care use in the current study was based on the usual source of health care classified as either primary care or secondary care provider only. Given the ongoing primary care-oriented healthcare reform in place in China, the examination of aspects such as the use of different models of primary care providers, total healthcare cost, drug prescriptions, and missed healthcare use due to cost to the patient would be useful in future research. Moreover, for the clinical management of patients with multimorbidity, the exploration of condition clustering patterns by socio-demographic risk strata may be important to ensure a tailoring of treatment strategies to need and improved processes of care.

## Conclusions

With the study samples drawn from the world’s largest developing country with a transitional healthcare system built on a social medical insurance system, we have provided information on the epidemiology of multimorbidity and its associated factors, which are, in general, similar to other developed countries. The growing burden and cost of multiple chronic diseases worldwide is likely to require a generalist, primary care-based response rather than increasing specialist care [54]. Along with continuing socio-economic development in China, developing a high quality primary care-based approach built on continuity, coordination and whole person care focusing on healthy lifestyle would appear to be a top priority, especially in light of the growing issue of multimorbidity due to the rapidly ageing population combined with the legacy of China’s one-child policy.

## Additional files

Additional file 1: Figure S1. Sampling framework in the three prefectures. Additional file 2: Table S1. Major variables measuring demographic, socio-economic, and lifestyle behaviours in the study. Additional file 3: Table S2. List of the chronic conditions included in the multimorbidity count. Additional file 4: Table S3. Comparison of study population with the national census population. Additional file 5: Figure S2. Number of chronic conditions by age group.

## References

[1] Yang GH, Kong LZ, Zhao WH, Wan X, Zhai Y, Chen LC, Koplan JP (2008). Emergence of chronic non-communicable diseases in China. Lancet.

[2] World Health Organization: *Preventing Chronic Diseases: A Vital Investment.* Geneva, Switzerland: 2005.

[3] Fortin M, Bravo G, Hudon C, Vanasse A, Lapointe L (2005). Prevalence of multimorbidity among adults seen in family practice. Ann Fam Med.

[4] Barnett K, Mercer SW, Norbury M, Watt G, Wyke S, Guthrie B (2012). Epidemiology of multimorbidity and implications for health care, research, and medical education: a cross-sectional study. Lancet.

[5] Stange KC (2012). In this issue: challenges of managing multimorbidity. Ann Fam Med.

[6] Dawes M (2010). Co-morbidity: we need a guideline for each patient not a guideline for each disease. Fam Pract.

[7] Yip W, Hsiao WC (2008). The Chinese health system at a crossroads. Health Aff (Millwood).

[8] Gallacher KI, Batty G, McLean G, Mercer SW, Guthrie B, May CR, Langhorne P, Mair FS (2014). Stroke, multimorbidity and polypharmacy in a nationally representative sample of 1,424,378 patients in Scotland: implications for treatment burden. BMC Med.

[9] Salisbury C (2012). Multimorbidity: redesigning health care for people who use it. Lancet.

[10] Starfield B, Shi LY, Macinko J (2005). Contribution of primary care to health systems and health. Milbank Q.

[11] Smith JP, Majmundar MK, National Research Council (U.S.) (2012). Aging in Asia: Findings from New and Emerging Data Initiatives.

[12] Chen Z (2009). Launch of the health care reform plan in China. Lancet.

[13] Liu Q, Wang B, Kong YY, Cheng KK (2011). China’s primary health-care reform. Lancet.

[14] Ministry of Health: *Healthy China Plan 2020.* Beijing, P. R. China: 2008.

[15] State Council: *Guidance on Developing Community Health Services in the Cities. No.10 Document.* Beijing, P. R. China: 2006.

[16] Wang HH, Wang JJ, Griffiths SM, Tang JL, Yeoh EK (2014). Developing Primary Care in China. Routledge Handbook of Global Public Health in Asia.

[17] Wang HH, Wong SY, Wong MC, Wei XL, Wang JJ, Li DK, Tang JL, Gao GY, Griffiths SM (2013). Patients’ experiences in different models of community health centers in southern China. Ann Fam Med.

[18] Timmins N, King Edward’s Hospital Fund for London, European Observatory on Health Systems and Policies (2013). The Four UK Health Systems: Learning from Each Other.

[19] Hu SL, Tang SL, Liu YL, Zhao YX, Escobar ML, de Ferranti D (2008). Reform of how health care is paid for in China: challenges and opportunities. Lancet.

[20] Bambra C, Gibson M, Sowden A, Wright K, Whitehead M, Petticrew M (2010). Tackling the wider social determinants of health and health inequalities: evidence from systematic reviews. J Epidemiol Community Health.

[21] Violan C, Foguet-Boreu Q, Flores-Mateo G, Salisbury C, Blom J, Freitag M, Glynn L, Muth C, Valderas JM (2014). Prevalence, determinants and patterns of multimorbidity in primary care: a systematic review of observational studies. PLoS One.

[22] Salisbury C, Johnson L, Purdy S, Valderas JM, Montgomery AA (2011). Epidemiology and impact of multimorbidity in primary care: a retrospective cohort study. Br J Gen Pract.

[23] National Bureau of Statistics of China: *The Sixth National Population Census.* Beijing, P. R. China: 2011.

[24] China International Urbanization Development Strategy Research Committee: *China Urbanisation Rate Investigation Report.* Beijing, P. R. China, 2011.

[25] Ministry of Health: *China Health Statistics Yearbook.* Beijing, P. R. China, 2011.

[26] The United Nations Statistics Division: *Designing Household Survey Samples: Practical Guidelines*, Volume 98. New York, USA, 2008.

[27] Center for Health Statistics and Information, Ministry of Health: *National Health Services Survey in China.* Beijing, P. R. China, 2008.

[28] Di Iorio CK (2005). Measurement in Health Behavior: Methods for Research and Education, Volume 1.

[29] Lauritsen JM, Bruus M (2008). EpiData Entry. A Comprehensive Tool for Validated Entry and Documentation of Data.

[30] Diederichs C, Berger K, Bartels DB (2011). The measurement of multiple chronic diseases-a systematic review on existing multimorbidity indices. J Gerontol A Biol Sci Med Sci.

[31] Payne RA, Abel GA, Guthrie B, Mercer SW (2013). The effect of physical multimorbidity, mental health conditions and socioeconomic deprivation on unplanned admissions to hospital: a retrospective cohort study. CMAJ.

[32] Huntley AL, Johnson R, Purdy S, Valderas JM, Salisbury C (2012). Measures of multimorbidity and morbidity burden for use in primary care and community settings: a systematic review and guide. Ann Fam Med.

[33] Hunt K, Adamson J, Galdas P, Kuhlmann E, Annandale E (2010). Gender and Help-Seeking: Towards Gender-Comparative Studies. The Palgrave Handbook of Gender and Healthcare.

[34] Malmusi D, Artazcoz L, Benach J, Borrell C (2012). Perception or real illness? How chronic conditions contribute to gender inequalities in self-rated health. Eur J Public Health.

[35] Marengoni A, Angleman S, Melis R, Mangialasche F, Karp A, Garmen A, Meinow B, Fratiglioni L (2011). Aging with multimorbidity: a systematic review of the literature. Ageing Res Rev.

[36] Kaneda T (2006). Chinas Concern over Population Aging and Health.

[37] United Nations: Department of Economic: *World Population Prospects: The 2004 Revision. Sex and Age Distribution of the World Population.* 2nd edition. New York, USA, 2005.

[38] Alcorn T (2013). China’s new leaders cut off one-child policy at the root. Lancet.

[39] Ryan A, Galvin R, Fortin M, Smith SM: *Lifestyle Risk Factors and Multimorbidity Risk: A Systematic Review [Research Protocol]. PROSPERO International Prospective Register of Systematic Reviews: PROSPERO 2014.* York, UK: CRD42014009593; 2014.

[40] Fortin M, Haggerty J, Almirall J, Bouhali T, Sasseville M, Lemieux M (2014). Lifestyle factors and multimorbidity: a cross sectional study. BMC Public Health.

[41] Mercer SW, Watt GC (2007). The inverse care law: clinical primary care encounters in deprived and affluent areas of Scotland. Ann Fam Med.

[42] Tang S, Tao J, Bekedam H (2012). Controlling cost escalation of healthcare: making universal health coverage sustainable in China. BMC Public Health.

[43] Li X, Shen JJ, Lu J, Wang Y, Sun M, Li C, Chang F, Hao M (2013). Household catastrophic medical expenses in eastern China: determinants and policy implications. BMC Health Serv Res.

[44] Xu Y, Wang L, He J, Bi Y, Li M, Wang T, Jiang Y, Dai M, Lu J, Xu M, Li Y, Hu N, Li J, Mi S, Chen CS, Li G, Mu Y, Zhao J, Kong L, Chen J, Lai S, Wang W, Zhao W, Ning G (2013). Prevalence and control of diabetes in Chinese adults. JAMA.

[45] Ezzati M, Vander Hoorn S, Lawes CM, Leach R, James WP, Lopez AD, Rodgers A, Murray CJ (2005). Rethinking the “diseases of affluence” paradigm: global patterns of nutritional risks in relation to economic development. PLoS Med.

[46] Muntner P, Gu D, Wildman RP, Chen J, Qan W, Whelton PK, He J (2005). Prevalence of physical activity among Chinese adults: results from the International Collaborative Study of Cardiovascular Disease in Asia. Am J Public Health.

[47] Diamond J (2003). The double puzzle of diabetes. Nature.

[48] Hussey PS, Schneider EC, Rudin RS, Fox DS, Lai J, Pollack CE (2014). Continuity and the costs of care for chronic disease. JAMA Intern Med.

[49] Rasella D, Harhay MO, Pamponet ML, Aquino R, Barreto ML (2014). Impact of primary health care on mortality from heart and cerebrovascular diseases in Brazil: a nationwide analysis of longitudinal data. BMJ.

[50] Wang HH, Wang JJ, Zhou ZH, Wang XW, Xu L (2013). General practice education and training in southern China: recent development and ongoing challenges under the healthcare reform. Malays Fam Physician.

[51] Parry J (2010). GP based primary care is only just starting to emerge in China. Brit Med J.

[52] Schoen C, Osborn R, Squires D, Doty M, Pierson R, Applebaum S (2011). New 2011 survey of patients with complex care needs in eleven countries finds that care is often poorly coordinated. Health Aff (Millwood).

[53] State Council: *Guiding Opinions of the State Council About the Pilot Urban Resident Basic Medical Insurance. No. 20 Document.* Beijing, P. R. China, 2007.

[54] World Health Organization: *The World Health Report 2008: Primary Health Care: Now More Than Ever.* Geneva, Switzerland, 2008.

