# Supplementary material for: Epidemiology of multimorbidity in China and implications for the healthcare system: cross-sectional survey among 162,464 community household residents in southern China
Source: BMC Med. 2014 Oct 23;12:188. doi: 10.1186/s12916-014-0188-0 (PMC4212117; doi:10.1186/s12916-014-0188-0)
Supplement: Additional file 1: Figure S1. — Sampling framework in the three prefectures. [file 12916_2014_188_MOESM1_ESM.doc]

**Supplementary Figure S1: Sampling framework in the three prefectures**

*The Sixth National Population Census. National Bureau of Statistics of China., 2011, http://www.stats.gov.cn/zgrkpc/dlc/yw/t20110428_402722384.htm.

Note: A multistage, stratified random sampling was adopted for selection of residential communities. In the first stage, all districts where community health centres (CHCs) were in place as primary care providers were selected as Primary Sampling Units (PSU). In the second stage, sub-districts within each PSU were randomly selected as Secondary Sampling Units (SSU). In the third stage, residential communities within each SSU were randomly selected as Tertiary Sampling Units (TSU). Households within TSU were randomly selected from the household lists obtained from the Community Neighbourhood Authority (a grass-roots administrative agency for household registration). All residents listed on the Household Register were sampled. The number of households selected (N=120) was calculated with the standard formula (Designing household survey samples: practical guidelines: United Nations. Statistical Division. United Nations Publications, 2008). Given that prefectures with larger total household residents tend to have smaller estimated household size, the number of sub-districts (n=4) and residential communities (n=2) to be randomly selected were the same across the three prefectures, in order to yield approximately equal number of household residents sampled. Randomisation procedure was done in PASW Statistics 18.0 (Chicago, Illinois, US).
