# Supplementary material for: Epidemiology of multimorbidity in China and implications for the healthcare system: cross-sectional survey among 162,464 community household residents in southern China
Source: BMC Med. 2014 Oct 23;12:188. doi: 10.1186/s12916-014-0188-0 (PMC4212117; doi:10.1186/s12916-014-0188-0)
Supplement: Additional file 2: Table S1. — Major variables measuring demographic, socio-economic, and lifestyle behaviours in the study. [file 12916_2014_188_MOESM2_ESM.doc]

**Additional Table S1: List of the chronic** conditions included in the multimorbidity count

| **Conditions** | **Mental/physical**  **health condition** | **Study population** | | **National sample*** |
| --- | --- | --- | --- | --- |
|  |  | **N** | **Prevalence (%)** | **Range of prevalence (%)** |
| Hypertension^†,§^ | Physical | 15,675 | 9.6 | 1.6-13.2 |
| Chronic painful condition^†^ | Physical | 7,797 | 4.8 | N/A |
| Inflammatory connective tissue disorders^†,§^ | Physical | 6,853 | 4.2 | 2.0-3.9 |
| Diabetes^†,§^ | Physical | 6,205 | 3.8 | 0.1-4.0 |
| Lipid disorder | Physical | 5,501 | 3.4 | N/A |
| Dyspepsia and gastroenteritis^†^ | Physical | 4,876 | 3.0 | 1.0-1.4 |
| Coronary heart disease^†,§^ | Physical | 3,351 | 2.1 | 0.8-4.4 |
| Chronic obstructive pulmonary disease^†,§^ | Physical | 2,694 | 1.7 | 0.7-1.2 |
| Stroke and cerebrovascular disease^†,§^ | Physical | 2,468 | 1.5 | 0.2-1.4 |
| Nephritis and chronic kidney disorder^†,§^ | Physical | 2,140 | 1.3 | 0.7-1.2 |
| Gallbladder / Spleen diseases | Physical | 1,598 | 1.0 | 0.4-0.7 |
| Peripheral vascular disease^†^ | Physical | 742 | 0.5 | 0.2-0.6 |
| Schizophrenia / bipolar disorder^†^ | Mental | 839 | 0.5 | 0.1-0.3 |
| Cancer^†,§^ | Physical | 810 | 0.5 | 0.1-0.5 |
| Multiple sclerosis / neurological disorder^†^ | Physical | 752 | 0.5 | 0.3-0.6 |
| Dementia^†,§^ | Mental | 691 | 0.4 | N/A |
| Bronchiectasis^†^ | Physical | 649 | 0.4 | 0.4-0.9 |
| Glaucoma / Cataract^†^ | Physical | 600 | 0.4 | 0.2-0.5 |
| Asthma^†^ | Physical | 485 | 0.3 | 0.2-0.3 |
| Chronic sinusitis^†^ | Physical | 495 | 0.3 | N/A |
| Viral Hepatitis^†^ | Physical | 456 | 0.3 | 0.1-0.2 |
| Chronic pharyngitis / Laryngitis | Physical | 380 | 0.2 | 0.1-0.2 |
| Diverticular disease of intestine^†^ | Physical | 325 | 0.2 | N/A |
| Thyroid disorders^†^ | Physical | 304 | 0.2 | N/A |
| Inflammatory bowel disease^†^ | Physical | 278 | 0.2 | 0.3-0.4 |
| Hearing loss/ Tinnitus^†^ | Physical | 228 | 0.1 | N/A |
| Blindness / Low vision^†^ | Physical | 208 | 0.1 | N/A |
| Psoriasis / eczema^†^ | Physical | 142 | 0.1 | 0.1-0.2 |
| Anaemia | Physical | 133 | 0.1 | 0.1-0.3 |
| Prostate disorders^†^ | Physical | 104 | 0.1 | 0.1-0.2 |
| Migraine^†^ | Physical | 97 | 0.1 | N/A |
| Chronic liver disease^†^ | Physical | 79 | <0.1 | 0.1-0.2 |
| Depression^†,§^ | Mental | 66 | <0.1 | N/A |
| Epilepsy^†^ | Physical | 58 | <0.1 | N/A |
| Anxiety & other stress related disorders^†^ | Mental | 48 | <0.1 | N/A |
| Parkinson’s disease^†^ | Physical | 40 | <0.1 | N/A |
| Irritable bowel syndrome^†^ | Physical | 37 | <0.1 | N/A |
| Constipation^†^ | Physical | 36 | <0.1 | N/A |
| Chronic tonsillitis | Physical | 36 | <0.1 | N/A |
| Anorexia / bulimia^†^ | Mental | 28 | <0.1 | N/A |
| **Overall prevalence of chronic diseases** |  |  | 23.8 | 20.0 |

*Data comes from China Health Statistics Yearbook (Ministry of Health. P.R.China, 2009) and National Health Services Survey, NHSS 2008 (N=177,501). The NHSS captured fourteen chronic conditions including: malignant neoplasm (cancer), diabetes mellitus, chronic rheumatic heart disease, angina pectoris, other forms of ischemic heart disease, pulmonary heart disease, hypertension, cerebrovascular disease, emphysema, chronic obstructive pulmonary disease (COPD), other forms of COPD, asthma, chronic liver disease and liver cirrhosis, and rheumatoid arthritis and disc disease [Center for Health Statistics and Information, Ministry of Health, P.R.China].

^†^Conditions listed by Barnett K, Mercer SW, Norbury M, *et al*. [Lancet 2012, 380:37-43].

^§^Conditions listed by Diederichs C, Berger K, Bartels DB. [J Gerontol a-Biol 2011, 66:301-311].
