# Supplementary material for: Epidemiology of multimorbidity in China and implications for the healthcare system: cross-sectional survey among 162,464 community household residents in southern China
Source: BMC Med. 2014 Oct 23;12:188. doi: 10.1186/s12916-014-0188-0 (PMC4212117; doi:10.1186/s12916-014-0188-0)
Supplement: Additional file 3: Table S2. — List of the chronic conditions included in the multimorbidity count. [file 12916_2014_188_MOESM3_ESM.doc]

**Additional Table S2: Comparison of study population with the national census population**

| **Variables** | **Study population, n (%)** | **General population, %** |
| --- | --- | --- |
|  | **(n = 162,464)** | **(national census)** |
| **Gender*****^†^** |  |  |
| Female | 78,972 (48.6%) | 48.73% |
| Male | 83,492 (51.4%) | 51.27% |
| **Age, years*****^†^** |  |  |
| 0-14 | 26,876 (16.5%) | 16.60% |
| 15-59 | 114,193 (70.3%) | 70.14% |
| **≥** 60 | 21,395 (13.2%) | 13.26% |
| **Education level^†^** |  |  |
| No education | 18,876 (11.8%) | 11.47% |
| Primary school | 39,023 (24.3%) | 26.78% |
| Secondary school | 81,779 (50.9%) | 52.82% |
| College and above | 20,918 (13.0%) | 8.93% |
| **N of household members on average^†^** | 3.02 | 3.10 |
| **Marital status^‡^** |  |  |
| Single | 35,111 (21.9%) | 16.4% |
| Married | 119,675 (74.5%) | 74.8% |
| Divorce | 1,239 (0.8%) | 1.4% |
| Widowed | 4,570 (2.8%) | 7.1% |
| **Employment status^‡^** |  |  |
| Unemployed | 27,994 (17.4%) | 16.6% |
| Employee | 101,020 (62.9%) | 66.9% |
| Retired | 16,346 (10.2%) | 9.6% |
| Student | 15,235 (9.5%) | 7.0% |
| **Medical insurance^‡^** |  |  |
| Insured | 134,890 (84.0%) | 86.1% |
| **Smoking^‡^** |  |  |
| Current smoker | 24,616 (15.3%) | 25.1% |
| **Alcohol consumption^‡^** |  |  |
| Seldom drinker | 141,482 (88.1%) | 87.5% |

Note: The prevalence on smoking in the national census was determined among population aged above 15 and above.

**p* value calculated from chi-square goodness of fit test is higher than 0.05 (*p* = 0.329 for gender; *p* = 0.398 for age).

^†^The categories are grouped according to the Sixth National Population Census.: National Bureau of Statistics of China., 2011

**^‡^**The categories are grouped according to the National Health Services Survey in China, 2008. Center for Health Statistics and Information, Ministry of Health, P.R.China
