# Supplementary material for: Epidemiology of multimorbidity in China and implications for the healthcare system: cross-sectional survey among 162,464 community household residents in southern China
Source: BMC Med. 2014 Oct 23;12:188. doi: 10.1186/s12916-014-0188-0 (PMC4212117; doi:10.1186/s12916-014-0188-0)
Supplement: Additional file 4: Table S3. — Comparison of study population with the national census population. [file 12916_2014_188_MOESM4_ESM.doc]

**Supplementary Figure S2: Number of chronic conditions by age group**
