# Supplementary material for: Epidemiology of multimorbidity in China and implications for the healthcare system: cross-sectional survey among 162,464 community household residents in southern China
Source: BMC Med. 2014 Oct 23;12:188. doi: 10.1186/s12916-014-0188-0 (PMC4212117; doi:10.1186/s12916-014-0188-0)
Supplement: Additional file 5: Figure S2. — Number of chronic conditions by age group. [file 12916_2014_188_MOESM5_ESM.doc]

**Additional Table S3: Major variables measuring demographic, socio-economic and lifestyle behaviours in the study**

| **Variables** |  |  |
| --- | --- | --- |
| **Income** |  | The income was calculated as monthly household income per head to take into consideration both the income level and the household size as people are living in the context of family. It was a proxy measure calculated by taking the self-reported total household income including wages, salaries, bonus and all types of benefits and pensions, divided by the number of total individuals living in the household. |
| **Education level** |  | The education level referred to respondents’ degree of education that received or the level of school that currently enrolled, whichever is the highest. |
| **Medical insurance** |  | The presence of any nation-wide social medical insurance, including basic medical insurance, new rural cooperative medical scheme, Medicaid scheme for lower socially-economic population, catastrophic disease insurance and other social medical insurance schemes. |
| **Chronic conditions** |  | Any chronic conditions included in the multimorbidity count that have been diagnosed or treated by any healthcare providers. |
| **Usual source of healthcare** |  | The healthcare provider where respondents considered as their usual contact of and frequent affiliation with care when a chronic disease appeared or reoccurred. Primary care referred to outpatient healthcare services provided in community health centres (CHCs), while secondary outpatient care referred to outpatient healthcare services provided by specialists in secondary- or tertiary-level hospitals. |
| **Physical activity** |  | The presence of physical activities referred to currently having involved in any vigorous physical exercises in which the heart beats faster and the breathing is heavier for at least 20 minutes in leisure time every week actively as regular lifestyle behaviour. |
| **Alcohol consumption** |  | Regular drinkers referred to those who were currently engaging in alcohol drinking for a total of more than 20 units (for male) or 15 units (for female) of drink consumption per week; or for four days or more within every week as regular lifestyle behaviour. |
| **Smoking** |  | Smoking behaviour referred to having smoked at least 100 cigarettes in one’s life time. Current smokers referred to those who were currently smoking tobacco on a consistent basis as regular lifestyle behaviour. |
| **Dietary preference** |  | Bland diet preference referred to having foods that were generally soft, less salty and fatty, and were not spicy as regular dietary habit. |
